# Horizontal and vertical movements of humpback whales inform the use of critical pelagic habitats in the western South Pacific

**DOI:** 10.1038/s41598-020-61771-z

**Published:** 2020-03-17

**Authors:** Solène Derville, Leigh G. Torres, Alexandre N. Zerbini, Marc Oremus, Claire Garrigue

**Affiliations:** 1 UMR ENTROPIE, IRD, 101 promenade Roger Laroque, 98848 Nouméa, New Caledonia; 2 Operation Cétacés, BP12827, 98802 Nouméa, New Caledonia; 3 Sorbonne Universités, UPMC Univ Paris 6, IFD-ED129, 4 Place Jussieu, Paris, 75252 France; 4 Geospatial Ecology of Marine Megafauna Lab, Marine Mammal Institute, Department of Fisheries and Wildlife, Oregon State University, 2030 SE Marine Science Drive, Newport, 97365 OR USA; 5 Marine Mammal Laboratory, Alaska Fisheries Science Center, 2725 Montlake Blvd E, Seattle, 98112 WA USA; 6 WWF France, Parc Forestier Michel Corbasson, BP692, 98845 Nouméa, New Caledonia

**Keywords:** Ecology, Ocean sciences

## Abstract

Humpback whales (*Megaptera novaeangliae*) are known for their nearshore distribution during the breeding season, but their pelagic habitat use patterns remain mostly unexplored. From 2016 to 2018, 18 humpback whales were equipped with depth-recording satellite tags (SPLASH10) to shed light on environmental and social drivers of seamount association around New Caledonia in the western South Pacific. Movement paths were spatially structured around shallow seamounts (<200 m). Indeed, two males stopped over the Lord Howe seamount chain during the first-ever recorded longitudinal transit between New Caledonia and the east coast of Australia. Residence time significantly increased with proximity to shallow seamounts, while dive depth increased in the vicinity of seafloor ridges. Most of the 7,986 recorded dives occurred above 80 m (88.5%), but deep dives (>80 m, max 616 m) were also recorded (11.5%), including by maternal females. Deep dives often occurred in series and were characterized by U-shapes suggesting high energy expenditure. This study provides new insights into the formerly overlooked use of pelagic habitats by humpback whales during the breeding season. Given increasing anthropogenic threats on deep sea habitats worldwide, this work has implications for the conservation of vulnerable marine ecosystems.

## Introduction

Seamounts are recognized as important pelagic ecosystems and a major biome in the open ocean^1^. Defined as isolated topographic elevations with summit depths at least 100 m above the seafloor^2^, seamounts affect ocean circulation and mixing, leading to nutrient upwelling, and stimulated primary production^3^. In the tropics, where pelagic waters are generally nutrient depleted, seamounts can form an “oasis of productivity”, which trigger trophic cascades attracting marine megafauna^4–6^. Hence, pelagic predators such as billfish, pinnipeds, seabirds, sharks and tuna have shown attraction to relatively shallow seamounts (<1000 m depth)^1^. Toothed whales (e.g., sperm whales, beaked whales, dolphins) are known to associate with seamounts^6–8^, presumably finding enhanced feeding opportunities over these seabed features. In contrast, seamount use by baleen whales has rarely been described^9^.

Humpback whales (*Megaptera novaeangliae*) were recently discovered to visit seamounts during the breeding season and spring migration period occurring in tropical and subtropical latitudes^10–13^. Humpback whales seasonally migrate from the polar feeding grounds where they spend the summer, to the tropical breeding grounds where they mate and give birth during the winter. While they must spend extended periods of time in the open ocean, their habitat use patterns have primarily been studied nearshore^14–21^. Only in the last decade has satellite telemetry provided the means to monitor humpback whale at the scale of their extensive movements. In the western South Pacific, humpback whales were found to visit shallow seamounts in the late breeding season^11^. The purpose of these seamount stop-overs was hypothesized to be related to breeding activities, resting, use as navigational landmarks, or supplemental feeding^11^.

In the western South Pacific, humpback whales inhabit a primarily pelagic environment, spread with islands, reefs and seamounts^22^. As a result, breeding grounds are structured into separate populations and sub-populations with varying degrees of connectivity^23–27^. The endangered Oceania population of humpback whales^23^ includes the sub-population wintering off New Caledonia (and labelled by the International Whaling Commission as sub-stock E2^28^), which occupies the most western region of the South Pacific^29^. This region is neighboured by the East Australian coast where sub-stock E1^29,30^ migrates. Hence, humpback whales of New Caledonia and Australia are separated by the Coral Sea, a vast pelagic space stretching over more than 1,500 km covered with seamount chains and ridges, where marine megafauna distribution is poorly understood.

Pelagic seamounts may form crucial, yet formerly overlooked habitats for humpback whales to congregate and aid in movement across vast distances in Oceania^11^. Moreover, understanding the role played by seamounts in the distribution and movements of humpback whales in the open ocean is a prerequisite to effective management at the scale of giant marine protected areas (MPA) such as the recently created “Natural Park of the Coral Sea”. Movement and dive tracking of humpback whales in breeding areas has to-date only been conducted during short periods^31–35^ or too late in the season^11,36–41^, providing data that lacks the ability to capture pelagic behaviours within wintering latitudes. Here, integrated, implantable satellite tags were deployed on humpback whales in New Caledonia offshore waters to record horizontal and vertical movements over long durations (weeks to months) in open waters during the breeding season and early migration stages. Humpback whale movements and diving are analysed in relation to seamounts to understand their unique association with these ecologically important pelagic features.

## Results

### Localized and regional movements

**Figure 1:**
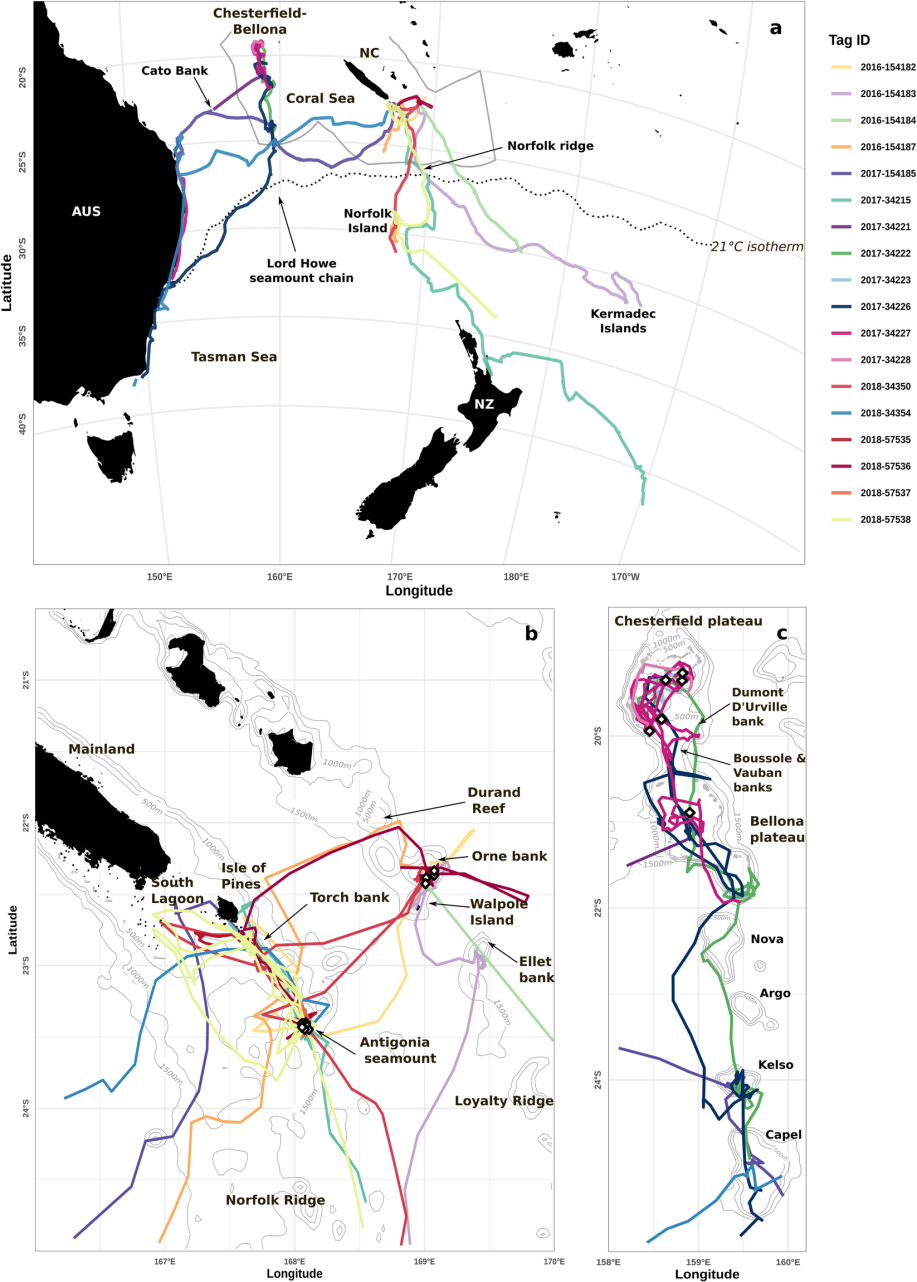
Satellite tracks recorded from 18 SPLASH10 tags deployed on humpback whales in New Caledonia. (**a**) Regional view (with southern borders of the Natural Park of the Coral Sea in grey) showing Australia (AUS), New Zealand (NZ) and New Caledonia (NC); (**b**) Zoom on Southeastern New Caledonia (Norfolk and Loyalty ridges); and (**c**) Zoom on the Chesterfield-Bellona coral reef complex and Lord Howe seamount chain. Grey lines represent 500 m isobaths up to 2000 m deep. Land is shown in black. The locations of tag deployments are shown with white diamond shapes. The breeding study region is demarcated by the 21 °C isotherm shown with a dotted line in (**a**).

Tagged whales included 7 males, 10 females and one individual of unknown sex. Five females were with a calf at the time of tagging, which were assumed to stay with their mother during the tracking duration. Whales were tracked for an average of 32.4 days (±s.d. 29.9), including an average of 20.2 days (±s.d. 16.3, Table 1) in the predefined breeding study region (Fig. 1a). They showed both localized (100 to 200 km wide) and regional movements (>1500 km wide). Once they left the breeding study region, whales were further tracked over their southward migration for an average of 12.3 days (max = 108.2 days).

**Table 1:** Summary of satellite tracking for the 18 humpback whales tagged with SPLASH10 tags (Wildlife Computers) in New Caledonia.

Year	ID	Sex/Status	Group type	Locality	Start	Tag duration (days)	Date leaving the breeding region	Within Breeding Region					
								Tag duration (days)	Minimum total distance (km)	# raw positions	# filtered positions	# dives recorded	Dive profiles recorded (hrs)
2016	154182	F/c	MCE	O	23/09/2016	9.8	—	9.8	809	56	49	165	2
	154183	F	P	O	24/09/2016	37	07/10/2016	13.9	971	133	126	736	5
	154184	F/c	MCE	O	23/09/2016	15.2	30/09/2016	7.7	571	32	30	80	3
	154187	M	MCR	O	24/09/2016	25.8	02/10/2016	8.8	1041	42	33	25	0
2017	34215	F	G3	A	24/07/2017	125.3	09/08/2017	17.1	1194	123	106	184	7
	154185	M	G3	A	24/07/2017	29	—	29	3429	242	206	975	36
	34222	F/c	MC	CB	22/08/2017	33.8	—	33.8	1907	203	187	555	18
	34223	—	P	CB	17/08/2017	6	—	6	390	42	35	110	5
	34226	F	S	CB	22/08/2017	46.7	21/09/2017	30.5	2705	206	169	465	5
	34227	F/c	MC	CB	18/08/2017	70.5	—	70.5	4858	450	386	1188	27
	34228	F/c	MC	CB	20/08/2017	4.8	—	4.8	279	25	24	100	6
	34221	F	P	CB	12/08/2017	5.8	—	5.8	496	5	6	25	0
2018	34350	F	C	A	17/07/2018	32.9	24/08/2018	19.9	1552	192	157	730	18
	34354	M	P	A	21/07/2018	45	12/08/2018	32.5	3915	173	87	205	6
	57535	M	G3	A	17/07/2018	8.8	—	8.8	500	94	81	249	7
	57536	M	G4	A	21/07/2018	21	—	21	1519	235	193	718	25
	57537	M	C	A	21/07/2018	10.8	—	10.8	617	154	131	715	24
	57538	M	G3	A	21/07/2018	54.7	21/08/2018	32.1	2618	344	277	761	32

#Stands for “number of”. The minimum total distance (km) is the along-the-path distance calculated from the CRW-interpolated tracks. Sex/Status: F = Female, F/c = Female with a calf, M = Male. Group type at the time of tagging: MC = Mother-calf, MCE = Mother calf escort, C = Competitive groups, G3 = Group of three adults, G4 = Group of four adults, P = Pair, S = Singleton. Locality: O = Orne bank, A = Antigonia seamount, CB = Chesterfield-Bellona archipelago. Date leaving the breeding region (see boundaries in Fig. 1a) is annotated “-” when the tag stopped emitting before the whale left the breeding region.

Humpback whales tagged in the seamounts and banks south of New Caledonia displayed localized movements between coastal and offshore shallow waters separated by 100 to 150 km (Fig. 1b). Among the 12 whales tagged in Orne bank and Antigonia seamount, three visited the coastal waters of the South Lagoon and four visited waters around the Isle of Pines. Connectivity between Orne bank and Antigonia was also revealed, as three whales out of the 12 tagged in this area visited both sites separated by 155 km (#154182, #57536, and #34350). Similar localized movements were observed for whales tagged in the Chesterfield-Bellona coral reef complexes. They displayed localized movements within these shallow plateaus before initiating their southward migration (Fig. 1c). While they spent some significant time in the sheltered waters of the inner reefs (mean = 10.2 days ±s.d. 9.8 days), they also visited shallow offshore features such as the Dumont D’Urville, Vauban and Boussole banks located between the Chesterfield and the Bellona plateaus.

Humpback whales leaving the waters south of the New Caledonia mainland also performed extensive regional movements following two main trajectories. Six whales moved south and southeast: three passed by Norfolk Island, one passed by New Zealand and two moved in the direction of the Kermadec Islands. Two males moved west (#154185 and #34354), stopped over the seamounts of the Lord Howe chain (for 7 and 3 days, respectively) and finally reached the East Australian coast around 25°S (Fig. 1a). Whale #154185 also stopped for 3 days around Cato Bank, an isolated bank with a small, emerged reef and islet located west of the Coral Sea.

In Chesterfield-Bellona, two females (#34222 with a calf, and #34226) could be tracked south of the coral reef complexes, and they both navigated through the Nova seamount, then spent time over the Kelso and Capel seamounts (10 days for #34222 and 4 days for #34226 over these two seamounts). Finally, three out of the six whales tagged in the Chesterfield-Bellona moved westward after leaving the Lord Howe chain. Two females (#34227 with a calf, and #34226) were tracked while migrating south along the East Australian coast in 2017, plus one male in 2018 (#34354).

### Diving behaviour in wintering latitudes

**Figure 2:**
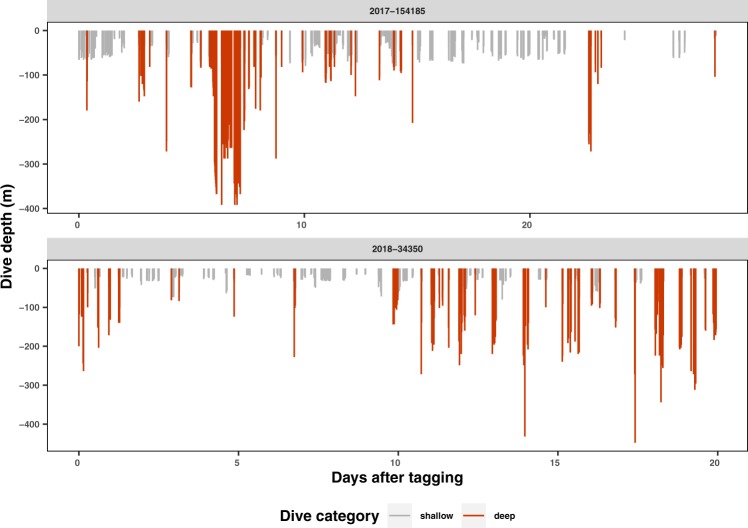
Dive depths through time for two whales tagged in New Caledonia (male #154185 and female #34350). The x-axis represents time from deployment in days. Each vertical bar represents a single dive event. Dives deeper than 80 m are shown in red.

Dive depths in the breeding region were mostly concentrated above 80 m deep (88.5% of dives, Table 2). A minority of dives occurred below 80 m (11.5%). The maximum dive depth of 616 m was reached by female #34226 east of the Bellona plateau in waters about 2,550 m deep. Deep dives below 80 m often occurred in series (Fig. 2), with 66% of deep dives occurring in a series of multiple (range: 2–20) deep dives. Deep dives were performed by all categories of individuals (Table 2), including females with a calf (max depth = 336 m).

**Table 2:** Summary of diving behavior recorded for the 18 humpback whales tagged with SPLASH10 tags (Wildlife Computers) in New Caledonia.

year	ID	Sex/Status	Locality	Dive depth (m)			Dive duration (min)			% dives >80 m
				mean	sd	max	mean	sd	max	
2016	154182	F/c	O	62.5	59.7	336	6.3	3.6	16.1	24.8
	154183	F	O	47.8	52.5	288	4.8	3.5	16.4	13.9
	154184	F/c	O	43.6	57.4	240	4.2	2.4	10.6	16.2
	154187	M	O	29.2	21.2	92	6.6	3.4	13.1	4
2017	34215	F	A	44	45.1	344	6.5	4.6	22	11.4
	154185	M	A	60	69.4	392	5.3	3.8	16.7	15.9
	34222	F/c	CB	47.7	40.6	288	5.6	3.3	18.4	15
	34223	_	CB	47.1	28	188	6	4.2	22.3	4.5
	34226	F	CB	50.1	52.9	616	6.9	4.7	22.1	10.5
	34227	F/c	CB	27.4	14.7	192	4.7	3	24.3	0.1
	34228	F/c	CB	28.2	16.7	74	6.6	3.1	13.8	0
	34221	F	CB	55.5	60.6	220	11.5	7.8	23.7	12
2018	34350	F	A	55.5	61.8	448	7.2	5.2	24.2	20.1
	34354	M	A	62.2	42.6	312	6.9	4.9	23.2	25.9
	57535	M	A	38.6	34.5	296	4.4	2.9	12.2	7.2
	57536	M	A	35.2	30.4	376	6.9	4.5	26.2	4.5
	57537	M	A	38	36.8	303	4.5	3.3	19	4.8
	57538	M	A	68.8	82	520	5.3	4.5	21.9	20.9

Sex/Status: F = Female, F/c = Female with a calf, M = Male. Locality: O = Orne bank, A = Antigonia seamount, CB = Chesterfield-Bellona archipelago. Diving behaviour is only considered within the predefined breeding region (see boundaries in Fig. 1a).

On average males dove to 51.1 m (±s.d. 59.2), females without a calf dove to 50.7 m (±s.d. 55.6), and females with a calf dove to 36.2 m (±s.d. 33.4). Sex and breeding status did not significantly influence dive depth (ANOVA with rank transformation: n.obs = 7,876, n.groups = 17, df = 2, F = 0.964, p = 0.405). Dive durations averaged 5.2 min (±s.d. 3.2) for females with a calf, 5.5 min (±s.d. 4.1) for males, and 6.3 min (±s.d. 4.7) for females without a calf. Dive duration did not significantly differ between females, females with a calf, and males (ANOVA with rank transformation: n.obs = 7,876, n.groups = 17, df = 2, F = 0.978, p = 0.401).

**Figure 3:**
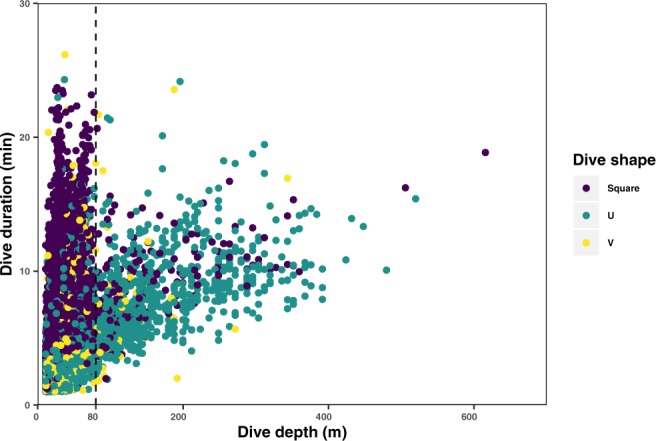
Relation between dive depth and duration for 18 whales tagged in New Caledonia (n = 7,986 dives). The dashed line delineates dives deeper than 80 meters. Dive shape (Square, U and V) are represented by color.

Dive depth and duration showed a positive non-linear correlation (n = 7,984, Spearman’s rho = 0.50, p = 2.2e-16). Based on dive shape, duration and depth, two categories of dives could be distinguished (Fig. 3). Deep dives below 80 m showed intermediate duration (mean = 8.3 min ±s.d. 3.3, max = 24.0 min) and primarily composed of U-shaped dives (76%). Shallow dives above 80 m and with long durations were primarily square-shaped (54%). V-shapes were the least common (6.2% of all dives), and were found both in deep and shallow dives.

**Figure 4:**
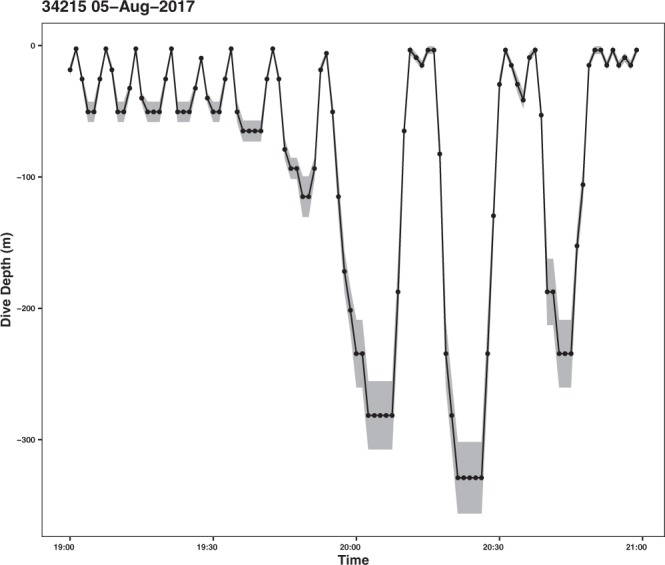
Example of a dive profile for the humpback whale #34215 (female), recorded at a frequency of one measurement every 75 seconds on August 5^th^, 2017. The grey ribbon shows the uncertainty of the depth measurement.

Dive profiles were recorded during 226 hours, spread over 170 separate dive bouts, representing an average of 14.1 hours (±s.d. 11.4) per individual. Among these dive profiles, 48 bouts representing 66 hours of recording contained at least one deep dive below 80 m. In these profiles, series of deep dives were observed and time spent at depth was evaluated (Fig. 4). On average, whales spent 2.5 min (±s.d. 1.5, max = 7 min) at maximum depth during deep dives recorded in the 48 profile bouts. Deep dives occurred in series of increasing depth in 44% of the dive profile bouts (Fig. 4), in series of decreasing depth in 8% of the bouts, and as stand-alone events in 21% of the bouts. For instance, the whale #34215 dove in a series of increasingly deep dives, from about 80 m to more than 300 m. Time spent at the bottom of the two deepest dives reached 5 minutes (Fig. 4).

### Pelagic movements in relation to seamounts

**Figure 5:**
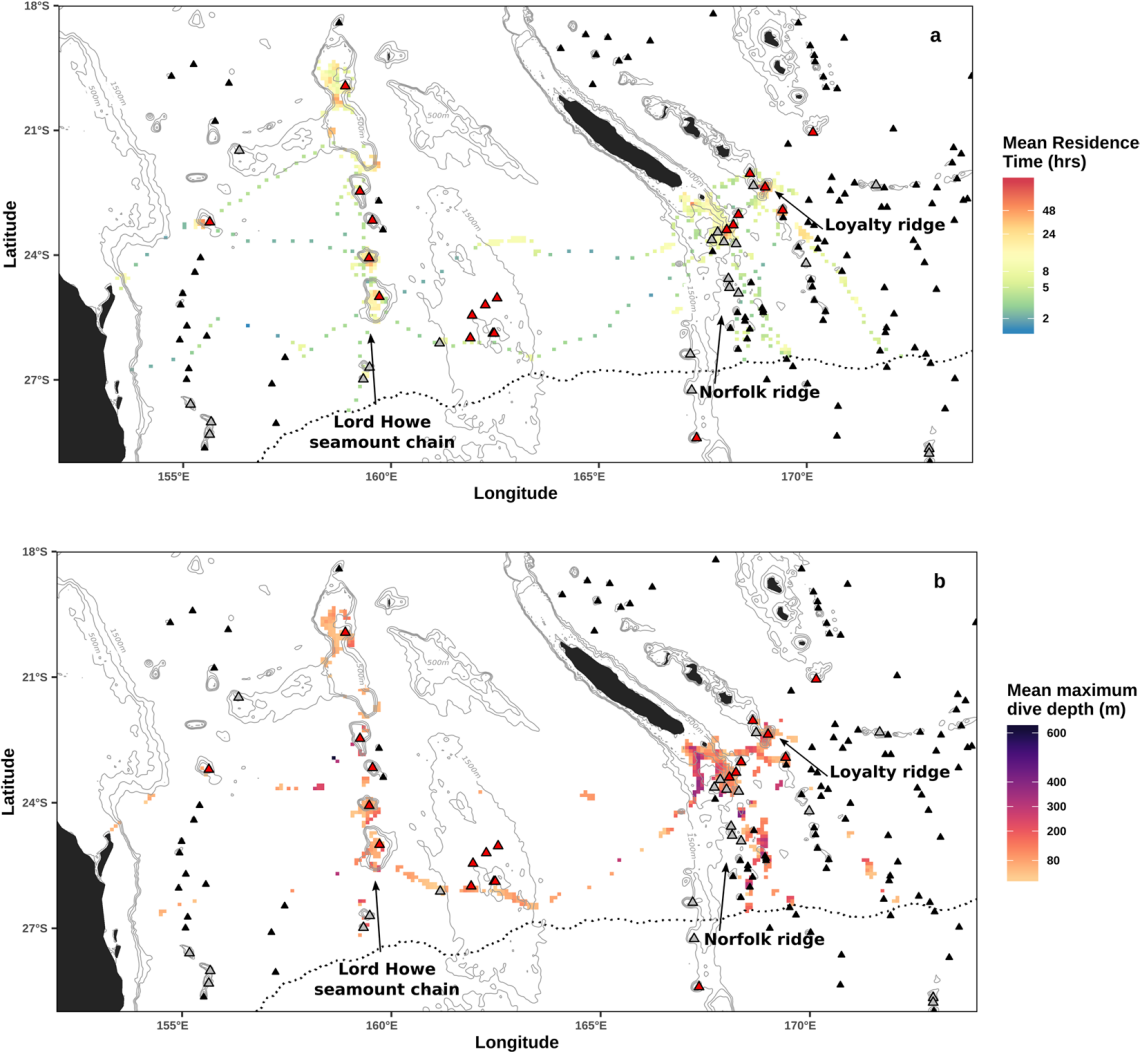
Pelagic horizontal and vertical movements averaged per individual over a grid of 10 km resolution. (**a**) Mean Residence Time (hrs) estimated from the CRW-interpolated tracks of 18 humpback whales. (**b**) Maximum dive depths (m) averaged for 16 humpback whales. Seamounts with varying depths are represented with triangles (Allain *et al*. 2008): shallower than 200 m (red), between 200 and 500 m (grey) and deeper than 500 m (black). Grey lines represent 500 m isobaths up to 2000 m deep. Land is shown in black. The breeding study region is demarcated by the 21 °C isotherm shown with a dotted line.

Out of 18 tagged whales, 16 individuals had dive events recorded in pelagic habitats. Many of the deep dives were recorded when whales were in the vicinity of the Lord Howe seamount chain, the Norfolk Ridge and the Loyalty Ridge (Fig. 5, and Supplementary Fig. S1). Diel phase significantly affected dive depth in pelagic habitats, as shallower dives were recorded at night (ANOVA with rank transformation: n.obs = 6,409, n.groups = 16, df = 1, F = 122.27, p < 0.0001; Supplementary Figs. S2 and S3).

**Figure 6:**
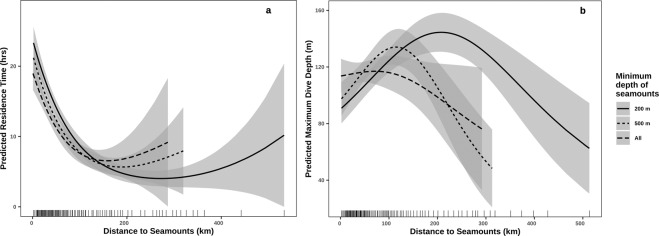
GAM predictions of horizontal and vertical movement of humpback whales in pelagic environment in response to the distance to seamounts. (**a**) Predicted Residence Time (hrs) from 18 CRW-interpolated tracks. (**b**) Predicted maximum dive depth (m). Rug plots illustrate the percentile distribution of the modelled distances to seamounts shallower than 200 m. The shaded ribbons represent approximate 95% confidence intervals.

Distance to seamounts of all depths significantly affected mean residence time (Table 3). Indeed, residence time increased when whales were close to seamounts (Fig. 6a), and this pattern was stronger for seamounts shallower than 200 m (deviance explained = 17.7%, Table 3). The mean maximum dive depth also appeared to be related to distance to seamounts although this relation was weak (Table 3). Distance to seamounts significantly affected dive depth when considering seamounts shallower than 500 m (deviance explained = 3.2%) and 200 m (deviance explained = 3.5%), but not when all seamounts were included (deviance explained = 0.5%). The average maximum dive depth was highest within 200 km of a shallow seamount <200 m (Fig. 6b).

**Table 3:** Summary of the Generalized Additive Models of mean maximum dive depths and mean residence time in relation to distance to seamounts.

	Distance to seamounts <200 m		Distance to seamounts <500 m		Distance to all seamounts	
	Deviance explained	Approximate significance	Deviance explained	Approximate significance	Deviance explained	Approximate significance
Mean residence time model	17.7%	Edf = 1.96, F = 57.8, p = <2e-16 ***	13.0%	Edf = 1.93, F = 44.1, p = <2e-16 ***	7.7%	Edf = 1.88, F = 27, p = 2.5e-11 ***
Mean maximum dive depth model	3.5%	Edf = 1.93, F = 10.2, p = 6.8e-05 ***	3.2%	Edf = 1.95, F = 8.15, p = 0.0004 ***	0.5%	Edf = 1.67, F = 0.79, p = 0.4

## Discussion

Using satellite tracking and dive recording, this study characterized vertical and horizontal movements of humpback whales in coastal and pelagic habitats of the Coral Sea during the breeding season. Tracking at a wide spatial scale allowed for a more comprehensive description of diving patterns and revealed a relatively high proportion of deep dives for adult whales of all social types, indicating unexpected energy expenditure, specifically around the Lord Howe seamount chain and the Norfolk ridge. A strong affinity for seamount habitats was further illustrated in horizontal movements. Finally, localized (100 to 200 km wide) and extensive regional movements (>1500 km wide) were revealed and shed light on the spatially-structured mating system of humpback whales.

Most studies of humpback whale diving patterns have been conducted in feeding areas^42–49^. A few studies have targeted the breeding season, but only over short tracking durations, generally less than a day^31–35^. In this study, we recorded a dive of 616 m, which appears to be the deepest dive ever recorded for an adult humpback whale, surpassing previously reported ‘deepest dives’ at 388 m (Antarctic Peninsula^48^), 392 m (West Greenland^47^) and 396 m (Hawaii^50^). Here, even mothers with a calf were found to dive as deep as 336 m. Calves of a few months old have been observed swimming down to 250 m in the Western Antarctic Peninsula^43^, but it is unknown whether the calves followed their mother on deep dives in this study.

Deep dives often occurred in series, and were U-shaped, resulting in prolonged time spent at the bottom of each dive, thus indicating large energy expenditure^44,48,49^. Several hypotheses could explain why humpback whales of all social types perform these energy-consuming deep dives in breeding regions. First, a scouting hypothesis could suggest that whales dive deep to gain orientation information during navigation between breeding grounds. Turbulences resulting from seafloor relief could constitute important cues for whales to localize their suitable shallow habitats or migration pathways^51^. No information is currently available to describe the mechanisms by which oceanographic stimuli may be sensed by baleen whales, but ocean circulation is postulated to be among the main sensory modalities used by baleen whales to locate prey^52^ and navigate^53^ at meso (10 km) to large scales (100 km). Deep dives may therefore serve a sensory and navigational role^54^, especially as shallow seamounts are shown to be the most attractive for humpback whales. Second, a social hypothesis could suggest that humpback whales dive deep to listen/communicate with conspecifics, or as a result of intra-specific interactions during competition for mating. Indeed, humpback whales in competitive groups have been observed with Crittercams displaying competitive behaviour at depth, up to 298 m^34^. Interactions within competitive groups composed of a female and several males could therefore explain some of the deep dives that occurred in the vicinity of recognized breeding grounds (e.g. Orne bank, Antigonia seamounts) where such competitive activities take place^55^.

The third hypothesis is that of opportunistic feeding. The successive deep U-shaped dives observed on several occasions are analogous to foraging dives of humpback whales and other rorquals^44,56^. Indeed, deep foraging dives often include a greater number of feeding lunges than during surface feeding, resulting in more time at depth^43,46^. For instance, blue whales (*Balaenoptera musculus)* and fin whales (*Balaenoptera physalus)* dive deeper and longer when feeding^57^. Although humpback whales are generally expected to fast during the breeding season^58,59^, occasional feeding outside feeding grounds has been reported in a few locations: in Samana Bay, Dominican Republic^60^ (19°N), off the coast of Brazil^61,62^ (19.5°S), in the Gulf of California^63^ (24.5°N), off the coast of Eden, east Australia^36^ (37°S) and in the New Caledonia South Lagoon (22°S, C. Garrigue pers. obs., a humpback whale was feeding at the surface on a fish school). Satellite tracking of humpback whales leaving their breeding grounds has also revealed Area Restricted Search interpreted as opportunistic feeding: off the Paracas coast, Peru^40^ (15 °S), in Magdalena bay, Baja California^64^ (25°N), over the Kermit-Roosevelt seamount, north of Hawaii^39^ (39°N), and over the Walters Shoal seamount, south of Madagascar^65,66^ (33°S). Although neither feeding nor defecation has ever been observed at the surface during offshore surveys in New Caledonia (C. Garrigue, pers. obs.), potential prey of the Euphausiid family has been found in these waters all year round^67^ and could provide opportunistic feeding opportunities. Interestingly, a small fraction of the micronekton biomass collected in New Caledonian offshore waters included *Euphausia mucronata*^67^, a krill species known to play a key role in the Humboldt Current System food chain, where it is consumed by fin whales^68^. In addition, in this study deep dives predominantly occurred during the day, a diel pattern that would be expected from feeding humpback whales following the vertical micronekton migration^48,69^. Finally, deep dives were predominantly observed over the seafloor ridges of the Coral Sea, but their relation to potentially enriched seamount waters was unclear. The low precision of the ARGOS positions (>50 km for “B” class^70^; composing 74% of the filtered positions in this study), and the relatively low frequency of data transmission (1 filtered position every 5.8 hours on average) impaired an accurate positioning of dive events with respect to small seamount features that extend over less than a dozen kilometres. Analysing diving behaviour at a finer spatial scale would help investigate the feeding hypothesis, as seamount slopes are often found to trigger primary productivity^1,3,4,71^, and could constitute opportunistic feeding spots for humpback whales in wintering latitudes.

Most survey effort to describe cetacean distribution and habitats is biased towards coastal waters^72^. In this study, implantable satellite tags allowed to investigate the use of pelagic waters by humpback whales during the breeding season, and confirmed the importance of shallow seamount habitat^11^, regardless of sex or breeding status of individuals. Indeed, several of the whales tagged in offshore waters (Antigonia or Orne bank) never stopped near a reef or a coast (seven out of 12) during the duration of tracking and remained in pelagic waters. Seamounts and banks that most attracted humpback whales included: Antigonia, Capel, Ellet, Kelso, Orne, and Torch (Fig. 1b,c). These seabed features have in common a relatively shallow depth at their summit (10 to 60 m), surrounding seabed of 1,500 to 2,000 m deep, and guyot shapes with flat summits of a surface ranging from 17 km^2^ (Ellet) to 1,800 km^2^ (Capel^73^). Humpback whales have been tracked during the breeding season over similar offshore features, such as Penguin Bank, in Hawaii^39,74^ or La Pérouse seamount in the western Indian Ocean^12^. Given their low latitude and/or proximity of these seamounts to known coastal breeding grounds, breeding activities were speculated^12^. In New Caledonia, *in situ* visual surveys conducted over the southeastern seamount region have confirmed that humpback whales display behaviours typically observed in breeding grounds: intense singing activity, competitive behaviour and nursing females^14,55^. Yet, it is intriguing to note that whales, including mothers with a calf, would prefer these unsheltered locations instead of selecting nearby coasts and lagoons to congregate^14^. Perhaps there are multiple benefits to these offshore seamounts.

What could be the benefits of pelagic seamount habitats for humpback whales? First, seamounts can represent landmarks in the open ocean. Both the shallow seabed feature itself, its geomagnetic signature^54^ and the peculiar turbulences that it triggers^1^ are likely to be detected by humpback whales. In this sense, seamounts are accessible and detectable areas for social aggregations during the breeding season. Second, it is possible that seamounts also act as “singing stages” for male humpback whales. These areas could be acoustically more suitable for singing males because they may be quieter than the surrounding pelagic environment and provide better sound propagation toward the open water^75^. As songs are likely to play a role in the spatial aggregation of individuals^76,77^, seamounts visited by great numbers of humpback whales could be acoustically attractive.

Humpback whales demonstrated localized and regional movements during the breeding season. A strong connectivity was observed at a relatively small scale among breeding aggregations of southeastern New Caledonia. Indeed, several whales repeatedly moved between breeding spots separated by 100 to 200 km. However, the extensive longitudinal movements (>1500 km) observed from east to west of the Coral Sea further question the scale at which the humpback whale may move during the breeding season. Longitudinal movements were characterized by transit-like parameters, typically observed during migration: low residence time, high swimming speeds and oriented travelling^10,11,36–38^. Two males moved between the well-known breeding grounds of southeastern New Caledonia, and the presumed breeding grounds of Capel and Kelso, located at a distance of about 850 km. Using the Lord Howe seamount chain as a stepping stone, they crossed the Coral Sea to reach the East Australian coast south of the E1 Great Barrier Reef breeding grounds^30^. Whether these animals were seeking mating opportunities over the Australian coast is unknown as the southerly limits to the East Australian breeding grounds are now thought to extend beyond the Great Barrier Reef^78^. So far, photo and genetic identifications have shown few resights between the New Caledonian breeding sub-stock E2 and the Australian migratory corridor supposed to be used by the breeding stock E1^25,79^. However, Valsecchi *et al*.^80^ suggested that some exchanges could result from differential migratory routes for males and females, specifically from extensive longitudinal movements of males during the breeding season. Further investigation into sex-specific movement dynamics in the region is warranted to understand these exchanges and the spatial scale at which the humpback whale mating system is organized. Nonetheless, extensive connectivity between aggregation sites within wintering latitudes challenges the longstanding view of humpback whale migration as a simple north-south migration with a single “final destination”.

## Conclusion

Satellite tag derived horizontal and vertical movements of humpback whales in the western South Pacific demonstrate that offshore shallow seamounts and banks play an important role in the spatially structured distribution of these whales during the breeding season. Several hypotheses are proposed to explain the unique diving behaviour and affinity for seamounts that were observed in pelagic waters. A deeper understanding of these offshore space use patterns has conservation implications at multiple scales. First, humpback whale population connectivity and dynamics could be reinterpreted in the light of this more comprehensive assessment of suitable nearshore and offshore breeding season habitats. Indeed, the intense use of pelagic waters located far from the coasts has consequences for estimating the structure of the endangered humpback whale breeding population of Oceania. Second, the intense use of a formerly overlooked habitat changes the understanding of exposure rates to threats for humpback whales during the breeding season. Third, the presence of an emblematic and endangered megafauna species over the seamounts of the western South Pacific has implications for the conservation of these vulnerable marine ecosystems^81^. Within the Natural Park of the Coral Sea, many seamounts are considered to be highly sensitive ecosystems^73^ with exceptional levels of biodiversity and endemism^82^. The present study therefore supports the potential for humpback whales to play the role of umbrella species of conservation to the benefit of seamount ecosystems in the western South Pacific.

## Methods

### Satellite tag deployment

A total of 18 SPLASH10 satellite tags (Wildlife Computers, Redmond, WA 98052, USA) were deployed between 2016 and 2018 in New Caledonia (Table 1). Tags were deployed in two offshore shallow areas (Antigonia seamount, n = 8, and Orne bank, n = 4) and one remote coral reef complex (the Chesterfield-Bellona archipelago, n = 6), in the Natural Park of the Coral Sea (Fig. 1a). Tags were implanted on adult whales, a few dozen centimetres forward of the dorsal fin, using a modified pneumatic line-thrower (ARTS, Restech) set to a minimum pressure of 10 bars^83^. Technical details about the tag deployments are presented in Supplementary Table S4. Tagged whales were photographed using digital cameras Canon 40D and 50D equipped with 70 × 300 mm or 100 × 200 mm lenses with magnifier 1.4. Tissue samples were collected with a crossbow with a specially adapted bolt^84^. Genomic DNA was extracted from these biopsy samples to identify sex^85^ and individuals^86^. After comparison with the New Caledonian humpback whale catalogs, photo-identification and genotyping allowed individual identification of tagged whales. Tagging and biopsy sampling were approved by the review board of the Department of Maritime Affairs under the government of New Caledonia (permits #2016-1391/GNC, #2017-1107/GNC and #2018-923/GNC). Fieldwork was carried out in accordance with the relevant guidelines and regulations.

### Marking the boundaries of the breeding region

The analysis of the tracking dataset was limited to a study region assumed to host mating, calving, nursing and early migration. It has been argued that breeding ground extents are restricted by water temperature rather than latitude. Rasmussen *et al*.^87^ found that breeding grounds from both hemispheres were included in an average SST range of 21.1 °C to 28.3 °C. Following this assessment, the climatology of austral winter SST was calculated for the region, using monthly remotely sensed SST products acquired for the months of July to October, from 2003 to 2014 with a spatial resolution of 1 km (MURSST, https://podaac.jpl.nasa.gov/dataset/MUR-JPL-L4-GLOB-v4.1). The average isotherm at 21 °C was calculated to delineate the southern boundary of the breeding study region (Fig. 1a, Table 1).

### Filtering and modelling satellite tracks

Data processing and statistical analysis were performed with R (version 3.4.4^88^). ARGOS locations were filtered to remove invalid locations of class Z, locations on land and locations implying unrealistically rapid movements (speed >18 km/h^89^). Whenever a track was interrupted for more than 72 hours, the track was considered to be constituted by several segments, subsequently projected in a Pacific-centered Mercator coordinate system and interpolated at one position every 6 hours with a Continuous-time Correlated Random Walk (CRW) model using the R *crawl* package version 2.1.1^90^. CRW model movement as a velocity process, characterized by two parameters: β, the velocity autocorrelation, and σ, the velocity variation. Using these models, the animal’s position can subsequently be predicted at any time, from the start to the end of the original track. The error on ARGOS positions was incorporated as the ellipses semi-minor and semi-major axis error, with deployment GPS positions included with ellipses logarithmic error set to 0. The β parameter was constrained between [−3, 4] bounds and was optimized using a Normal distribution prior with mean −0.15 and standard deviation 1.5. The σ parameter was left unconstrained and was optimized from a start value of log(10).

The distances covered were calculated along the crawl-interpolated track segments, within the previously identified breeding study region. Residence time was calculated along the crawl-interpolated tracks to assess movement type. Residence time is the total amount of time spent, both backward and forward, within a virtual circle (of radius *ρ*) centered on a given location, provided the animal did not move out of the circle for more than a time threshold (τ). Residence time therefore provides an integrative measure of space use^91^ and may reveal Area Restricted Search when animals slow down and display more sinuous paths as a result of a spatially-restricted activity (e.g., resting, feeding, or interacting with conspecifics). Area Restricted Search behaviour is scale-dependent, a pattern that can be tested using varying radii *ρ* in the residence calculation. Here, residence time was calculated in a radius *ρ* of 1, 5, 10 or 20 km (with a time threshold τ of 12 hours) for each tagged individual. The log-transformed variance of the residence time values was averaged across individuals in order to determine the best study scale^92^. The 10 km radius was found to maximize the variance of residence time and was selected for further analysis.

### Diving behaviour analysis

Diving behaviour analysis was limited to dives recorded while the humpback whales were in the breeding study region initially identified. For every dive event greater than 11 meters in depth and 1 min in duration, the SPLASH10 tags recorded three parameters: dive depth (maximum depth reached during dive, in meters), dive duration (in minutes) and dive shape. Dive depth is recorded by SPLASH10 tags as an interval (mean = 0.76 m ± s.d. 1.27) from which the median depth value was extracted. On very rare occasions (0.08% of dives), the wet/dry sensor of the tag may not have detected the surfacing event following a dive, resulting in aberrant values of dive duration (max = 62 min). Based on the distribution of outliers, dives >30 min were filtered out. Dive depth and duration were analysed at the tagged population scale, with all tagged individuals pooled together to describe the overall vertical movement characteristics within the breeding region. Based on the relation between dive depth and duration, dives were categorized into two classes: deep dives >80 m, and shallow dives between 11 and 80 m. Dive depths and durations were compared between males, females, and females with a calf, using one-way repeated measures ANOVA (i.e. within subject effect) with a rank transformation. Finally, dive shapes were used to infer behavioural modes after they were classified into three categories depending on the time spent at the bottom of the dive (i.e. below 80% of the maximum dive depth reached for a given dive): 50% of the dive duration for square-shaped dives, 20–50% for U-shaped dives, and less than 20% for V-shaped dives. Tags were also set up to record dive profiles during a period of 24 hrs, every 7 days (in 2016) or every 3 days (in 2017 and 2018). Dive profiles record the whale’s depth at a frequency of 75 s, which allowed a finer analysis of humpback whale behaviour at depth.

### Seamount effect on movement

The geographic positions of dives were estimated using the CRW models from each track segment. The *crwPredict* function from the R *crawl* package predicted dive position based on the time at which the dive occurred. Preliminary analysis showed that the positional error associated with predicted dive positions was positively correlated with the time elapsed between the dive and the most recent ARGOS filtered position recorded by the tag (longitude error: Pearson’s r = 0.71, t = 89.2, df = 7,984, p < 2.2e-16; latitude error: Pearson’s r = 0.76, t = 104.8, df = 7,984, p < 2.2e-16). In order to remove potentially mispositioned dive events, all dives recorded more than 6 hours away from an ARGOS position were removed from further analysis. Track and dive positions occurring in “sheltered” waters of the East Australian coast, the New Caledonian lagoons and the Chesterfield-Bellona plateaus were excluded to focus on humpback whale movements in pelagic waters (Supplementary Fig. S5). Dive depths in pelagic waters were compared between night and day time (using a 6 a.m./6 p.m. cut-off), using a one-way repeated measures ANOVA (i.e. within subject effect) with a rank transformation.

Pelagic movement characteristics were averaged over 10 km resolution grids. Residence time was averaged per grid cell for each tagged whale, then individual residence time grids were averaged together. The maximum dive depth was calculated per grid cell for each tagged whale, then individual dive grids were averaged together. Gridded residence time and maximum dive depth were modelled as a function of distance to the closest seamount using Generalized Additive Models (*mgcv* R package, version 1.8–23, GAM^93^). The positions and depths of seamounts were obtained from a Pacific database^94^. Seamount depths were validated within the New Caledonian economic exclusive zone using local bathymetric charts at 500 m resolution^95^ (whenever the local charts indicated shallower summit depths than the Pacific charts, the former values were used). As seamount summit depth has been identified as an important factor of attraction for cetaceans^6,8^, distance to seamounts was calculated in three ways: distance to seamounts of all depths, distance to seamounts shallower than 500 m, and distance to seamounts shallower than 200 m. Both the mean residence time and mean maximum dive depth were modelled as Gaussian response variables with a log link function. The smoothed effect of distance to seamounts was optimized by Restrictive Maximum likelihood and cubic smoothing splines with basis size limited to 3 to prevent overfitting^96^. The performance of models was assessed by the proportion of deviance explained^97^.

## Supplementary information

Supplementary information.
