# Supplementary material for: Horizontal and vertical movements of humpback whales inform the use of critical pelagic habitats in the western South Pacific
Source: Sci Rep. 2020 Mar 17;10:4871. doi: 10.1038/s41598-020-61771-z (PMC7078318; doi:10.1038/s41598-020-61771-z)
Supplement: Supplementary file 1 — Supplementary information. [file 41598_2020_61771_MOESM1_ESM.pdf]

Horizontal and vertical movements of humpback whales inform the use of critical pelagic habitats in the western South Pacific

Derville Solène[1,2,3,4]*, Torres Leigh G.[4], Zerbini Alexandre[5], Oremus Marc[6], Garrigue Claire[1,2]

[1] UMR ENTROPIE, (IRD, Université de La Reunion, CNRS, Laboratoire d'excellence-CORAIL), 101 promenade Roger Laroque, BPA5, 98848 Nouméa Cedex, New Caledonia

[2] Operation Cétacés, BP12827, 98802 Nouméa, New Caledonia

[3] Sorbonne Universités, UPMC Univ Paris 06, IFD-ED129, 4 Place Jussieu, 75252 Paris Cedex 05, France

[4] Geospatial Ecology of Marine Megafauna Lab, Marine Mammal Institute, Department of Fisheries and Wildlife, Oregon State University, Newport, HMSC, 2030 SE Marine Science Drive, Oregon 97365, USA

[5] Marine Mammal Laboratory, Alaska Fisheries Science Center, Seattle, Washington, WA, USA

Supplementary Figures and Tables

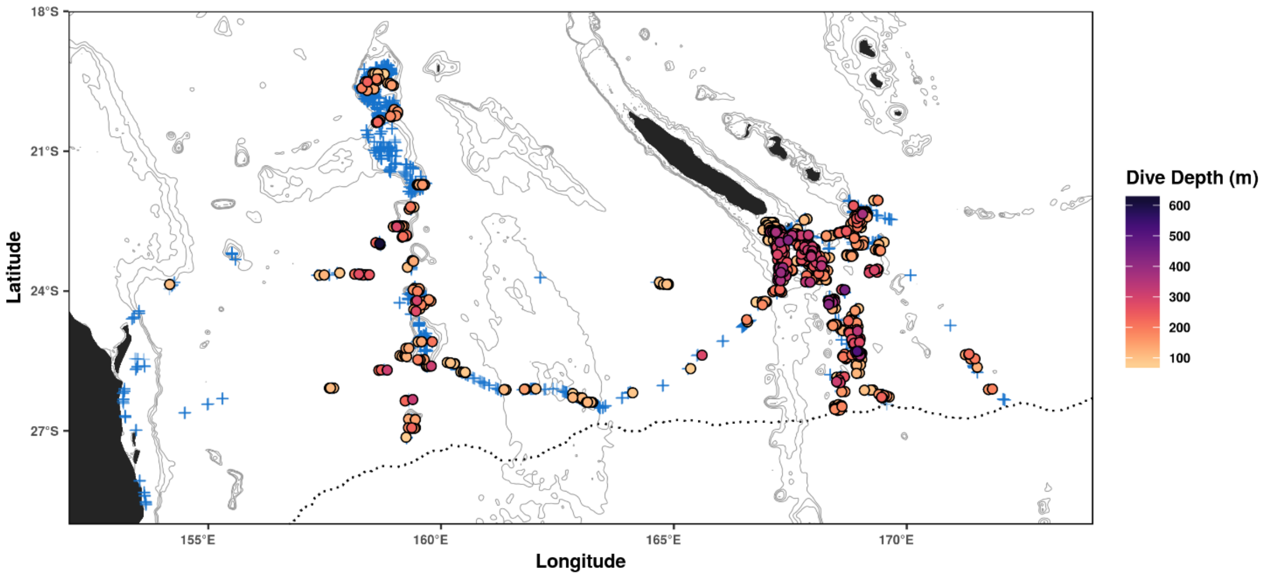

Figure S1: Geographic position estimated for dives recorded from 18 humpback whales tagged in New Caledonia (n = 7,712 dives). Dives deeper than 80 meters are represented with filled circles. Shallower dives are represented with blue crosses. Grey lines represent 500 m isobaths up to 2000 m deep. Land is shown in black. The breeding study region is demarcated by the 21°C isotherm shown with a dotted line.

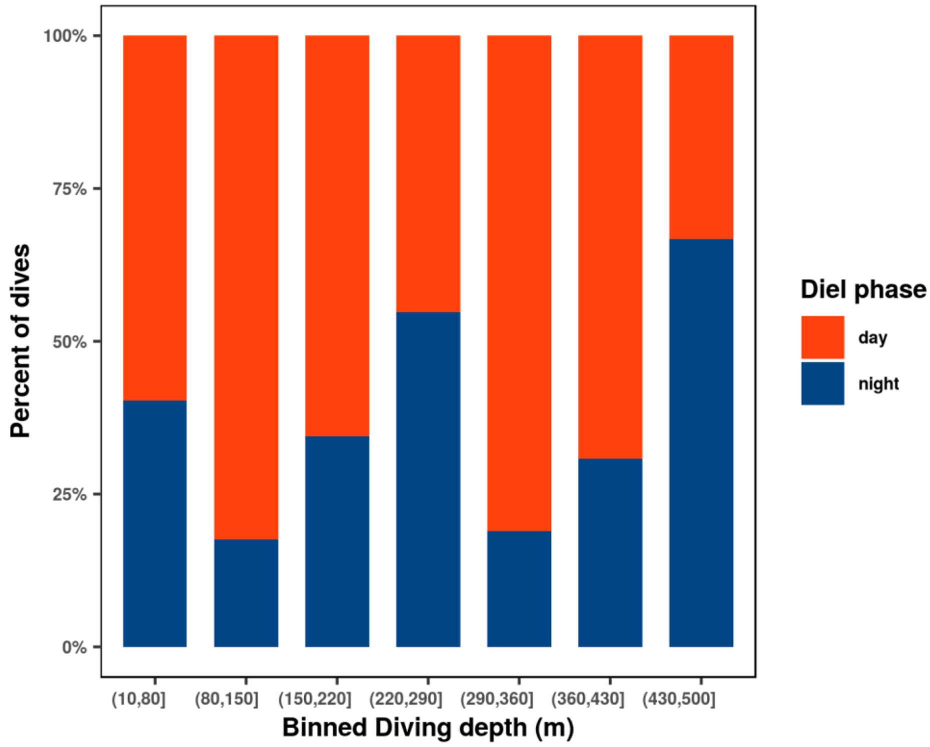

Figure S2: Dives recorded as a function of depth (%;all individuals pooled together, n = 6,409 dives) during the day (red) and night (blue)

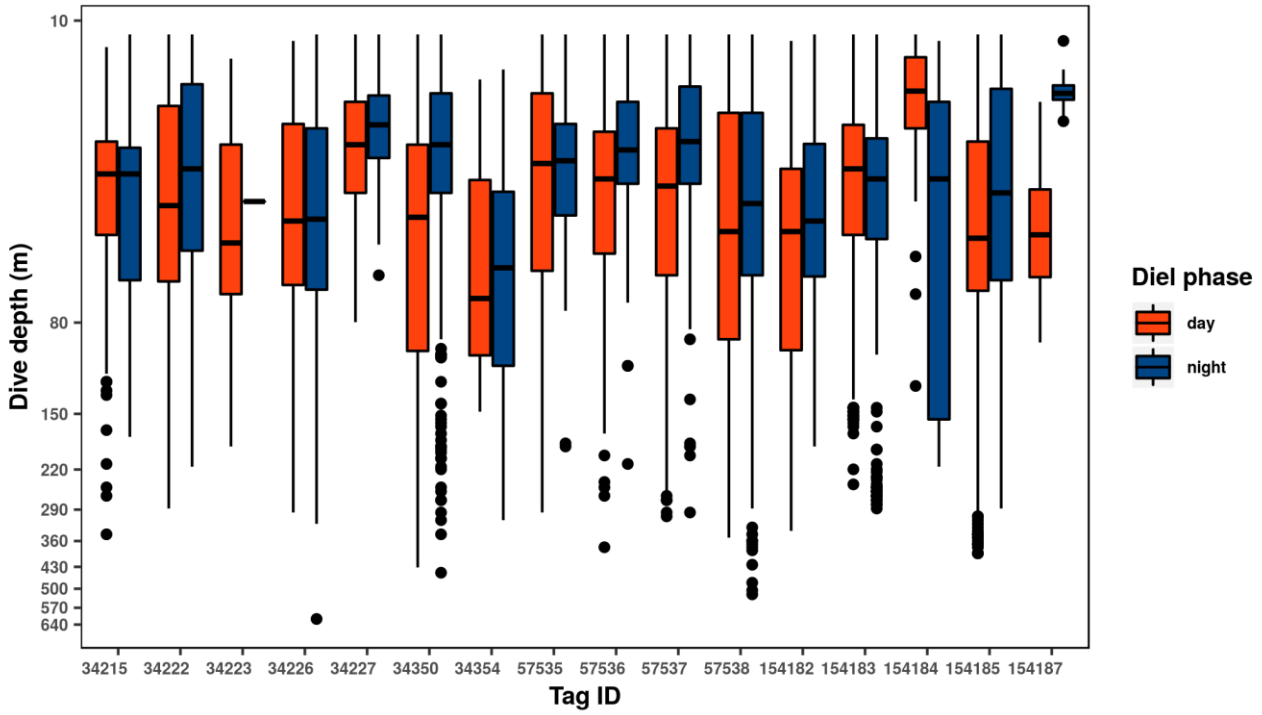

Figure S3: Depth of dives recorded at night or during the day per individual tracked in the pelagic breeding region (n = 6,409 dives).

Table S4: Technical details about tag deployments conducted between 2016 and 2018 in New Caledonia. Incomplete tag implantation varied from 3 to 10 cm of the tag's length not being implanted.

ID	Tag total duration (days)	Latitude at deployment	Longitude at deployment	ARTS pressure (bars)	Tag implantation
2016-154182	9.8	-22.3854	169.0127	10	incomplete
2016-154183	37	-22.3665	169.0738	10.5	complete
2016-154184	15.2	-22.4265	169.0076	10	incomplete
2016-154187	25.8	-22.3362	169.0748	10.5	complete
2017-34215	125.3	-23.4003	168.0863	10	incomplete
2017-154185	29	-23.4217	168.0764	10	complete
2017-34222	33.8	-19.3574	158.8189	10	incomplete
2017-34223	6	-19.2689	158.8242	10	complete
2017-34226	46.7	-19.3512	158.6364	11	complete
2017-34227	70.5	-19.8067	158.5894	10	complete
2017-34228	4.8	-19.9434	158.4567	10	incomplete
2017-34221	5.8	-20.8925	158.9035	10	incomplete
2018-34350	32.9	-23.4064	168.0629	10	incomplete
2018-34354	45	-23.4472	168.1079	11	incomplete
2018-57535	8.8	-23.4138	168.0851	10	incomplete
2018-57536	21	-23.4465	168.0765	12	complete
2018-57537	10.8	-23.4073	168.0540	12	complete
2018-57538	54.7	-23.4314	168.0568	12	complete

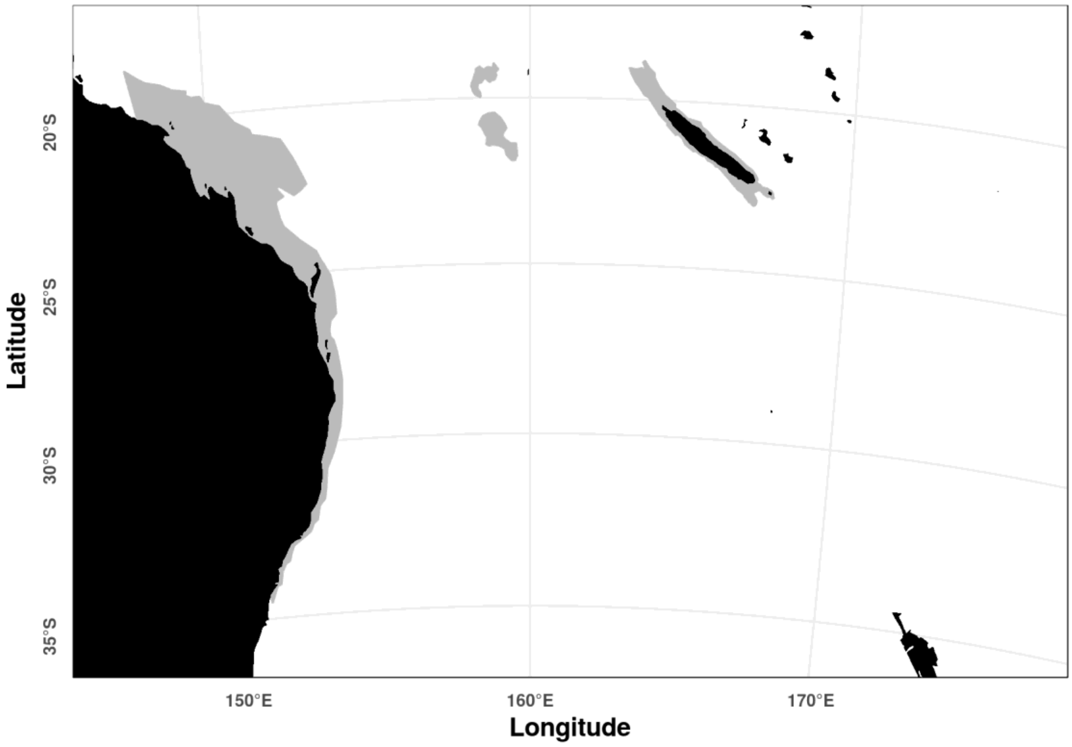

Figure S5: Map of sheltered waters excluded from the pelagic habitat movement analyses. Sheltered (coastal or lagoon) waters on the East Australian coast, New Caledonian lagoon and Chesterfield-Bellona lagoon were manually delineated (grey polygons).
